# Concurrent Multiple Myeloma and Metastatic Osteosarcoma: A Case Report and Literature Review

**DOI:** 10.7759/cureus.1634

**Published:** 2017-08-31

**Authors:** Ashley Ramirez, Vincent Grekoski, Michael Valente, Fiona Tissavirasingham, Alessandra Fahey, Nicole Guevara, John W Stelzer, M. Joe Ma, Jeremy Burt

**Affiliations:** 1 Diagnostic Radiology, Florida Hospital-Orlando; 2 College of Medicine, University of Central Florida; 3 Pathology, Florida Hospital-Orlando

**Keywords:** multiple myeloma, osteosarcoma, rare concurrent diagnosis, computed tomography, aggressive primary bone tumor

## Abstract

Multiple myeloma (MM) and osteosarcoma (OS) are two common bone malignancies, however, the simultaneous occurrence of both primary bone tumors in the same patient has not been reported in the United States to date. We present a unique case in which both malignancies present concurrently in a 72-year-old man. Results of spinal magnetic resonance imaging (MRI), radiographic skeletal survey, and hematological workup established the initial diagnosis of MM. Approximately three months later, the patient was admitted with severe right hip pain and shortness of breath and was evaluated with computed tomography (CT) of the right hip, abdomen, pelvis, and chest, revealing an osseous mass with a “sunburst” pattern in the right hip, and several calcified nodules in the lungs. Subsequent wedge resection and histological evaluation of the lung nodules confirmed the diagnosis of metastatic OS to the lungs, with a presumptive diagnosis of primary OS of the right hip. The clinical findings and imaging characteristics in this case are presented. Two similar cases found in the literature are also briefly discussed. The findings of this case report suggest that, in rare instances, MM patients with sclerotic bone findings may have a concurrent diagnosis of OS.

## Introduction

Multiple myeloma (MM), also known as Kahler’s disease, originates from the bone marrow and is the most common primary bone malignancy in the United States [1]. Patients commonly experience symptoms such as fatigue, severe bone pain, pathological fractures, osteoporosis, and neurologic deficits from spinal cord compression [2-3]. The resulting destructive bone loss of MM is due to an increase in bone resorption and a decrease in bone formation [2]. Radiographically, MM typically presents with lytic “punched-out” skeletal lesions; however, primary sclerotic manifestations occur in 3% of cases, some with a “sunburst” pattern [4].

Osteosarcoma (OS) is the second most common histologic form of primary bone malignancy, affecting mostly children and adolescents [5-6]. While OS is more prevalent in younger generations, 10% of cases present in the elderly, particularly in the seventh and eighth decades [7]. Although the incidence of OS within the United States is only 1%, OS is associated with a high mortality rate [5-6]. Patients with metastatic OS have an especially poor prognosis exhibiting an overall survival rate of 30% within five years; pulmonary metastasis, specifically, is often diagnosed, occurring in 40-50% of OS patients [6-7]. Classic radiographic features of OS include periosteal reactions manifesting as a Codman’s angle appearance and/or a “sunburst” pattern [8].

While both MM and OS are commonly occurring primary bone tumors, they rarely have been reported to occur simultaneously. In this case report, we present the first documented case of a combined diagnosis of MM and primary bone OS in the United States. Radiologic and histologic features pertaining to the case are emphasized with the intention of expanding the understanding of the concurrence of these malignancies. Given that, on rare occasions, MM can present with sclerotic bone lesions that overlap with the radiological features of OS, the findings of this study suggest that sclerotic bone lesions in MM patients may warrant further workup in order to correctly diagnose coexisting OS.

## Case presentation

**Figure 1:**
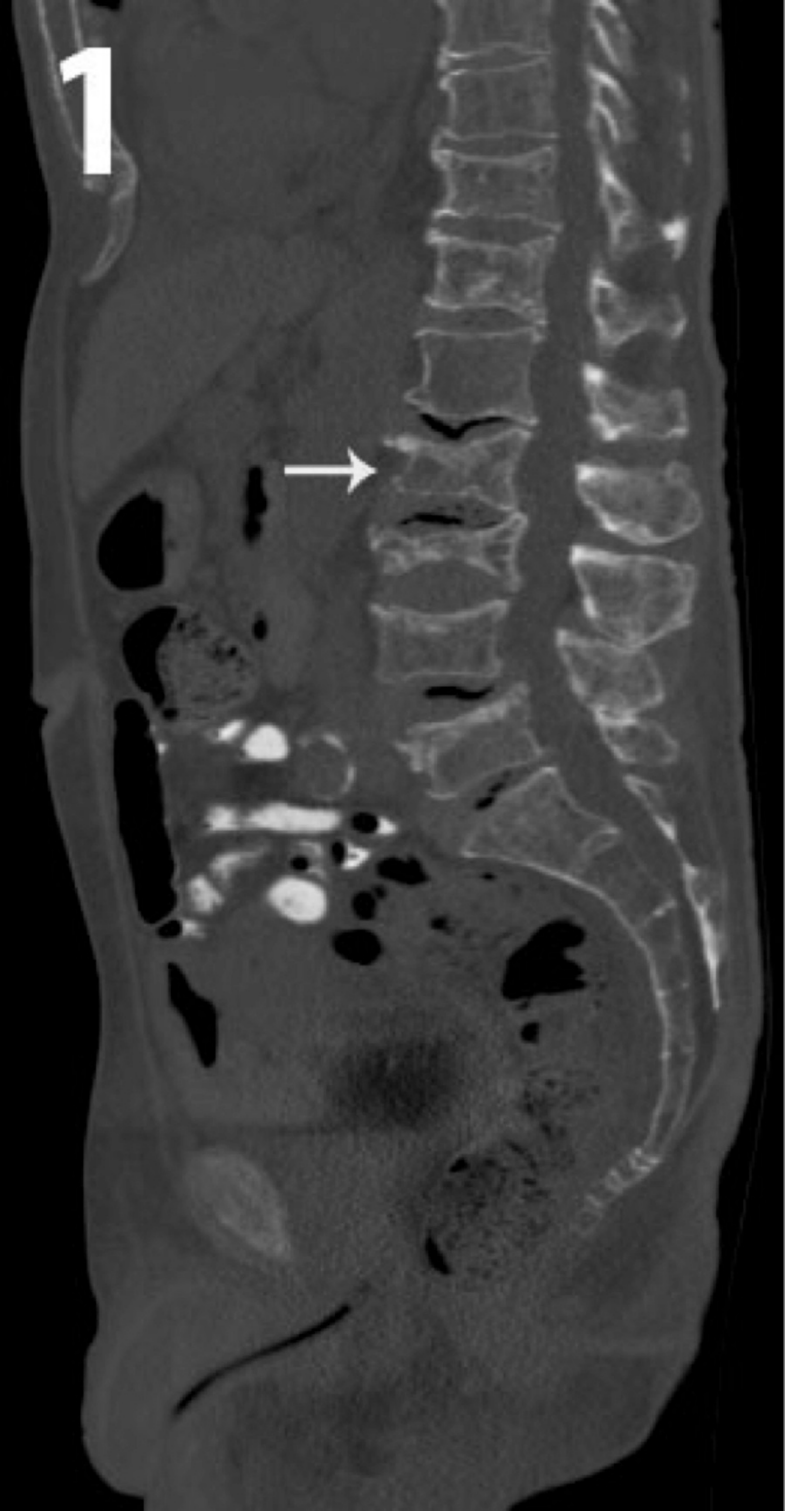
Sagittal CT image of the abdomen and pelvis using bone window levels. There is infiltration of the spine at multiple levels by multiple myeloma resulting in pathologic compression fractures (white arrow). CT- Computed Tomography

A 72-year-old male presented to our institution for orthopedic consultation following a visit to his primary care physician with a complaint of approximately five weeks of progressive bilateral lower back pain without a history of trauma. The pain was described as sharp with movement and constantly dull at rest with a rating of 4/10 on the VAS (Visual Analog Scale) pain scale. On physical examination, there was tenderness over L1 and L2, but no deficits in lower extremity sensation, motor strength, or reflexes. Computed tomography (CT) of the lumbar spine without contrast was performed and mild compression fractures of L2 and L3 were identified, likely subacute and non-healed (Figure 1). No soft tissue masses were noted. Abnormal marrow signal intensity was also seen compatible with an infiltrative marrow process such as MM. One week later, a follow-up magnetic resonance imaging (MRI) of the thoracic spine with and without contrast showed a 1.5 cm enhancing lesion in the right pedicle of T8, as well as a diffuse infiltrative process of the marrow. These results prompted a referral to hematology to confirm the diagnosis of MM.

Lab tests were performed by the hematology consultant to evaluate for the presence of MM. A serum protein electrophoresis showed gamma globulins reduced to 0.9% with an abnormal protein of 0.4 g%. The immunoglobulin studies showed a 0.34 g% monoclonal component of lambda light chains. Two samples of kappa and lambda light chains were elevated. One sample showed elevated lambda light chains to 335 mg/dL, giving a ratio of 0.6. The other sample showed elevated lambda light chains to 11,463 mg/dL. Chemistries were abnormal, with an elevated BUN (blood urea nitrogen) at 42 and creatinine at 1.98. Quantitative immunoglobulin studies were normal.

Due to the concerning results of the blood work, a radiographic bone survey was performed. It revealed innumerable small lytic lesions scattered throughout the axial and appendicular skeleton, all likely secondary to MM. The patient was subsequently put on a combination of bortezomib (Velcade) and dexamethasone for treatment, which worked well, decreasing lambda light chains from 11,463 mg/dL to 49.16 mg/dL on repeat lab work approximately 10 weeks later.

**Figure 2:**
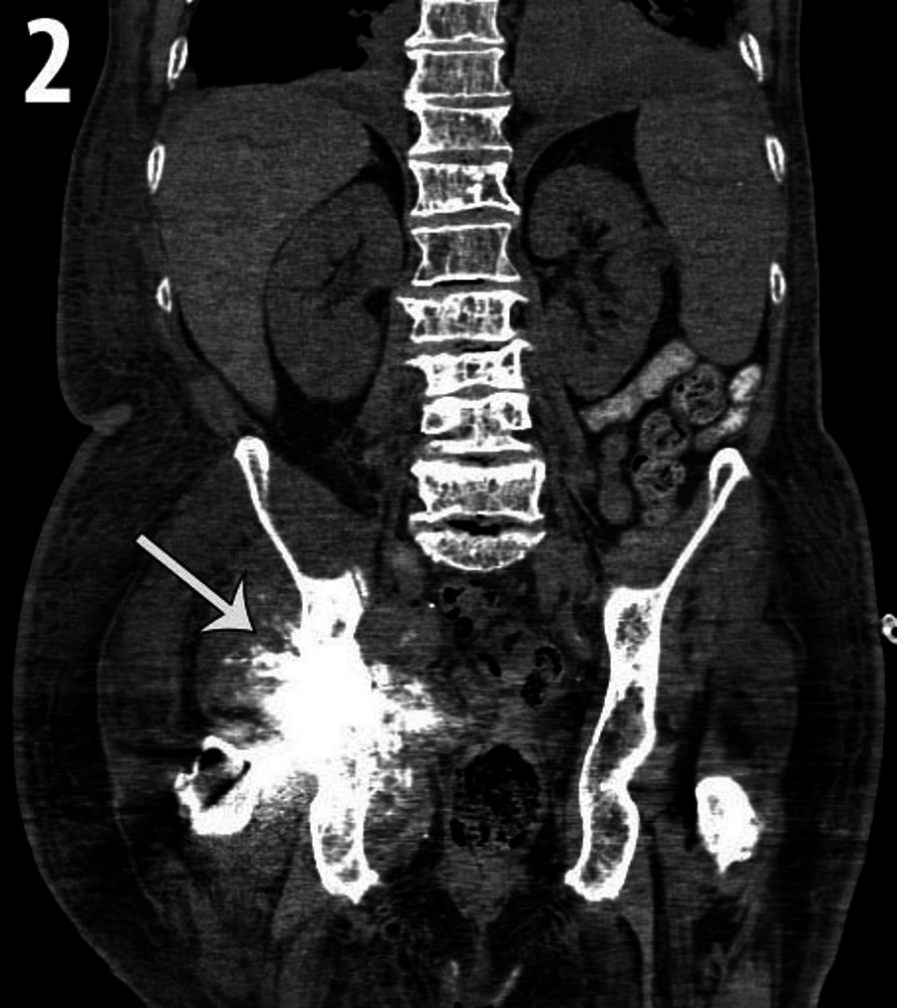
Coronal CT image of the abdomen and pelvis using soft tissue window levels. A destructive soft tissue mass is centered in the right acetabulum with a typical “sunburst” pattern of osteoid matrix (white arrow). Diffuse infiltration of the spine with myeloma is also noted. CT- Computed Tomography

**Figure 3:**
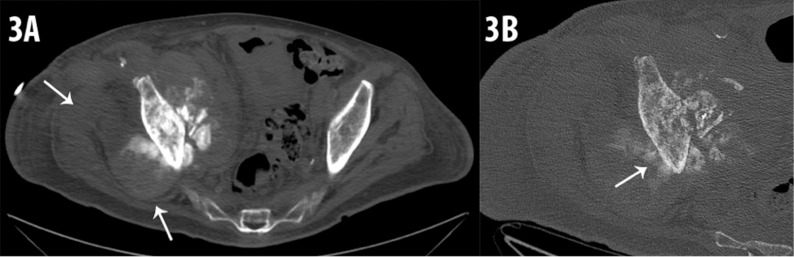
(A) Axial CT image of the pelvis. There is a soft tissue mass centered in the right acetabulum with dense sclerosis within the iliac bone, osteoid matrix and aggressive extension into the adjacent iliacus and gluteal musculature (white arrows). (B) High resolution axial CT with magnification of the right acetabulum. The destructive nature of the tumor within the cortex of the iliac bone is clearly defined. Osteoid matrix within the soft tissue component (white arrow). CT- Computed Tomography

The patient was admitted approximately three months after the myeloma diagnosis for extreme right hip pain and CT scan of the right hip, abdomen, and pelvis was performed. The study showed a right total hip arthroplasty and an osseous mass extending from the right acetabulum and right iliac crest. The mass had a “sunburst” pattern (Figure 2). The mass was partially ossified with soft tissue components (Figure 3A-3B).

**Figure 4:**
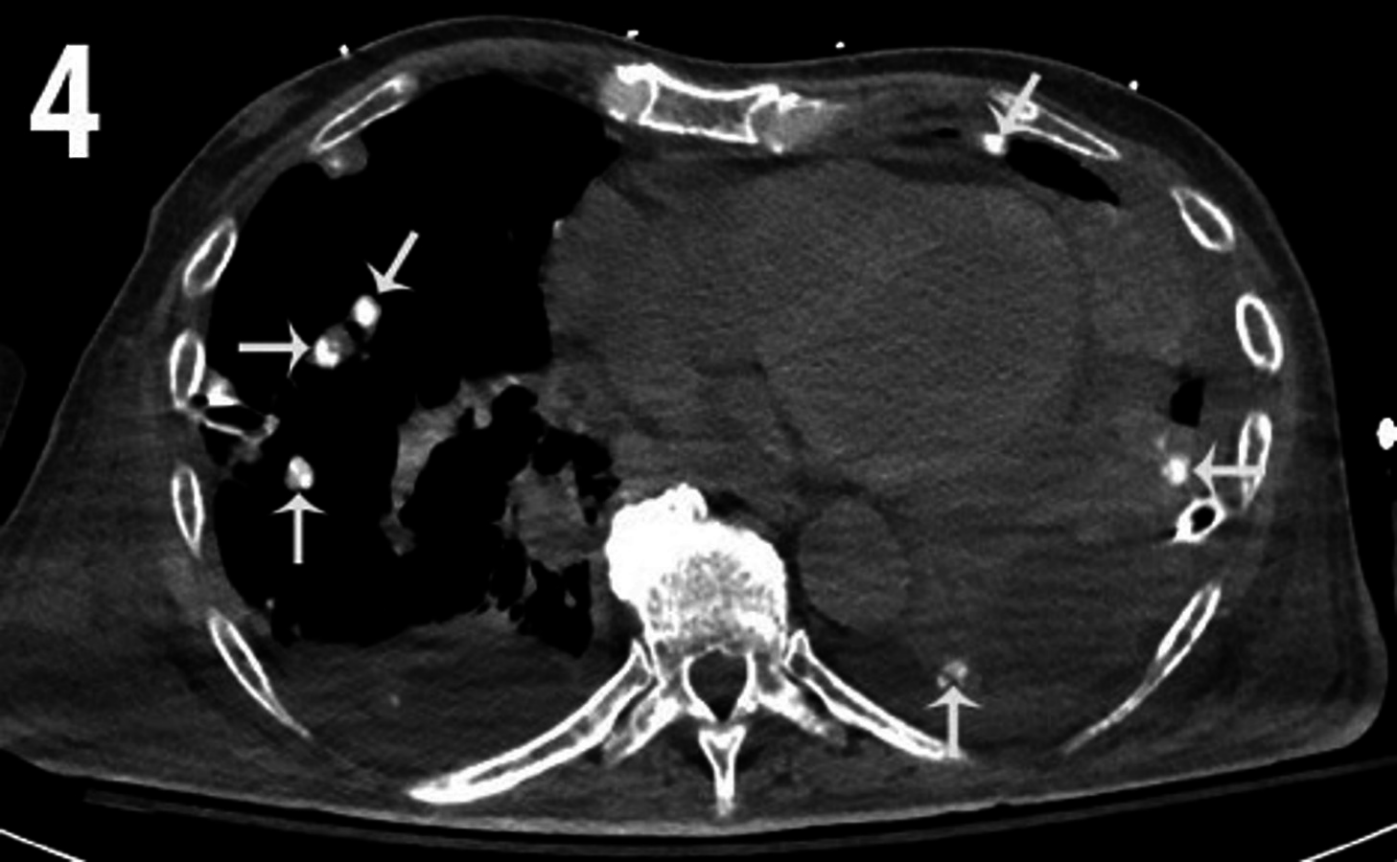
Axial CT image of the chest using soft tissue window levels. Multiple calcified bilateral lung nodules (white arrows). There are also noncalcified, pleural masses in the left cardiophrenic angle. Incidental note of small pericardial and pleural effusions. CT- Computed Tomography

**Figure 5:**
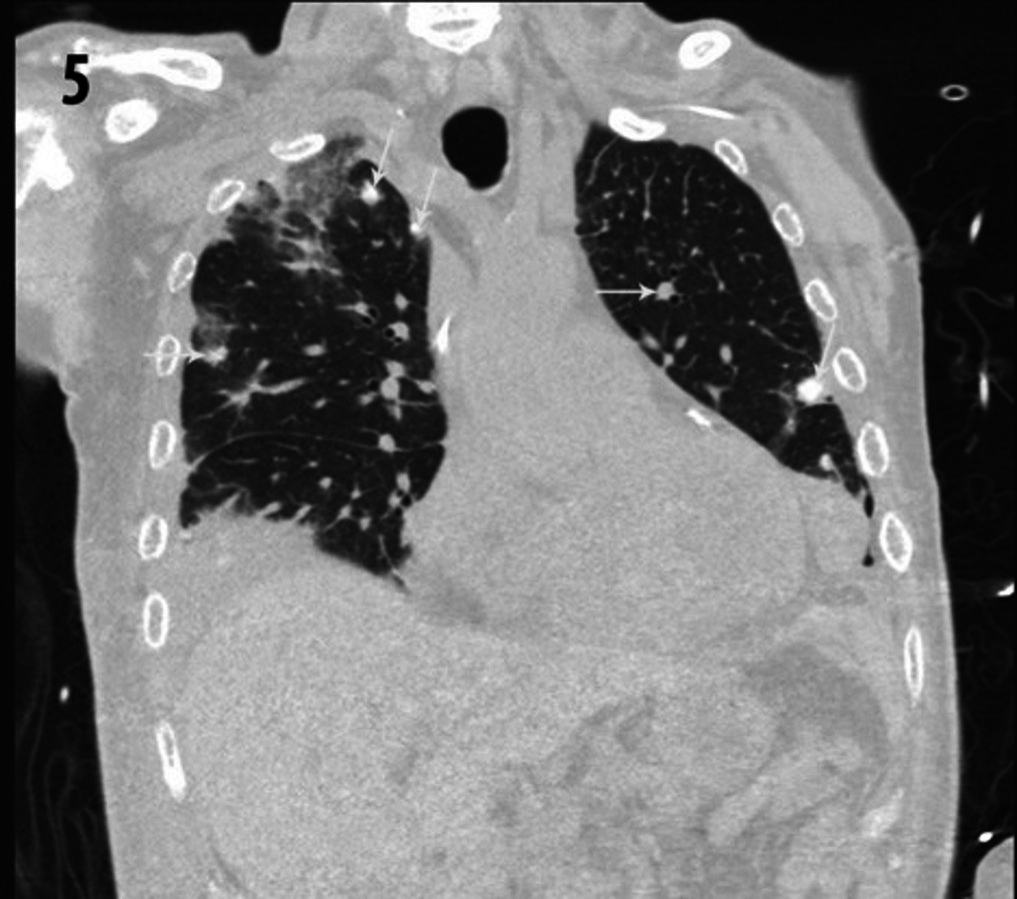
Coronal CT image of the chest using lung window levels. Multiple calcified pulmonary nodules in a random distribution (white arrows) compatible with hematogenous spread of osteosarcoma metastases. CT- Computed Tomography

Over the next few weeks, in addition to persistent severe right hip pain, the patient developed progressive shortness of breath, cough, and fever. Multiple CT scans of the chest over several weeks showed progressively worsening infiltrates, effusions, and mid and lower lung nodules, with most nodules showing calcifications (Figures 4-5). Metastatic OS from the right pelvis was suspected. Wedge resections of the left lower lobe and left upper lobe were obtained and histopathologic assessment was performed on the obtained tissue.

**Figure 6:**
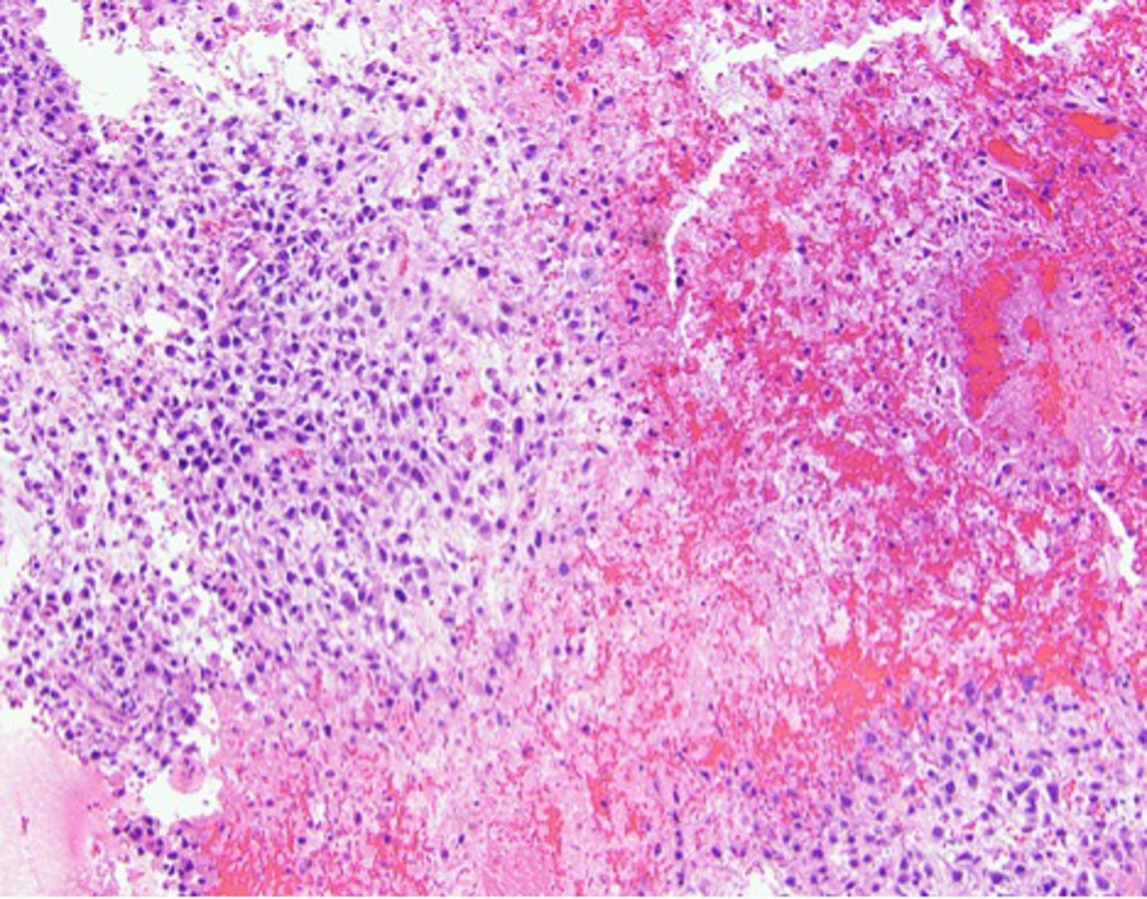
Low-power (100x, H&E stain) view of resected lung tumors shows fragments of viable neoplastic cells between large areas of hemorrhagic necrosis. This view shows a moderately cellular tumor consisting of loosely arranged polygonal cells. H&E - Hematoxylin and Eosin

**Figure 7:**
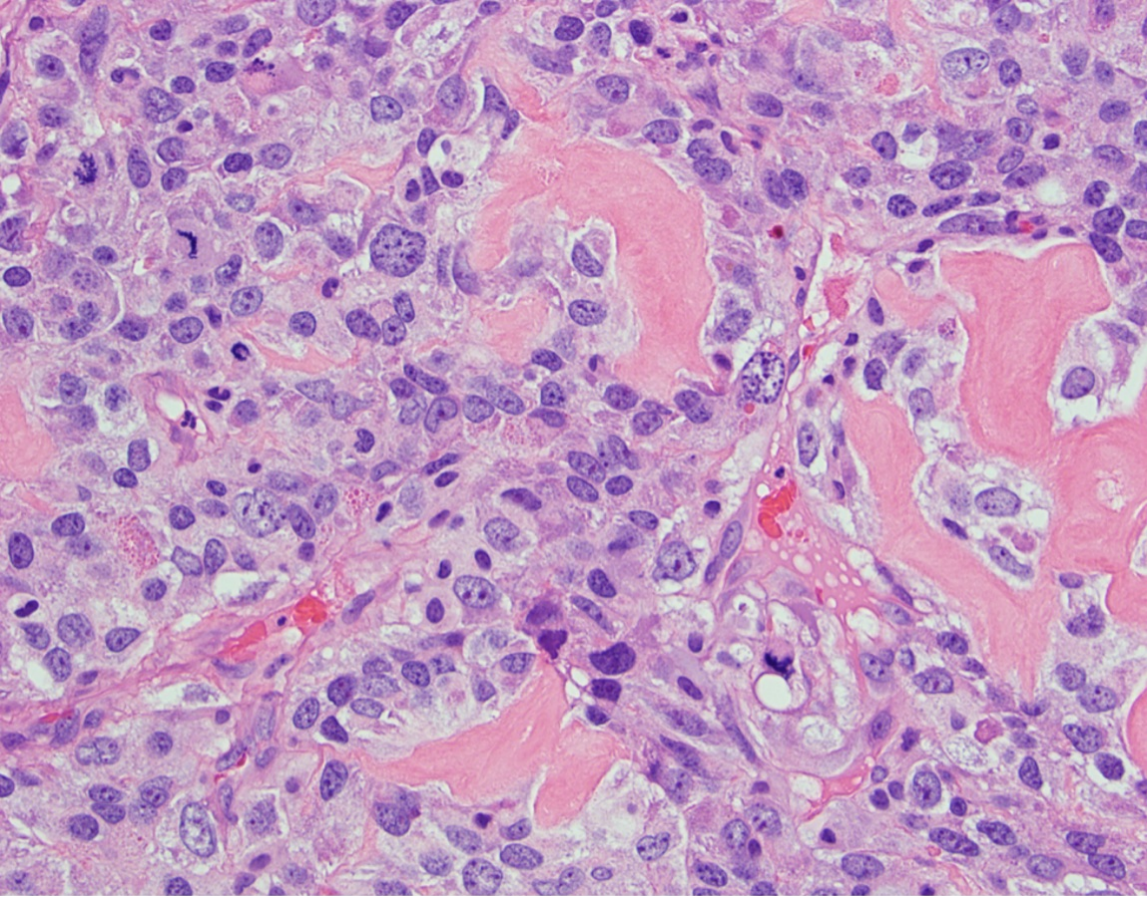
High-power (400x, H&E stain) view of neoplastic cells shows marked nuclear atypia, a brisk mitotic activity and, focally between cells, deposition of eosinophilic matrix consistent with malignant osteoid. H&E - Hematoxylin and Eosin

The resected tumors revealed identical histopathology. Figure 6 demonstrates areas of hemorrhagic necrosis and sheets of viable tumor cells that display irregularly shaped, variably sized, hyperchromatic, and sometimes vesicular nuclei. Varying amounts of pale eosinophilic cytoplasm without brown granular pigment, mucinous vacuoles, or cytoplasmic keratinization are also visualized (Figure 7). Architecturally, the tumor cells do not form glands, cysts, papillary fronds or any formed patterns. There are also curved or irregularly shaped deposits of eosinophilic extracellular matrix consistent with malignant osteoid (Figure 7). Immunohistochemical stains showed no immunoreactivity to four keratin markers (AE1, Cam 5.2, 34betaE12, CK5/6), S100, SOX10, calretinin, Wilms tumor-1, carcinoembryonic antigen (CEA), thyroid transcription factor-1 (TTF-1), or MOC-31. These overall findings were diagnostic of metastatic osteogenic sarcoma.

## Discussion

While both OS and MM are not uncommon, the concurrent presence of both malignancies is extremely rare. Only two other case reports of concurrent OS and MM are present in the literature [9-10]. One case report described a patient with MM developing a primary uterine OS with metastasis to the lungs and left bronchus [9]. The second case, reported in Europe, discussed a patient with MM who was treated with radiation and chemotherapy and subsequently developed primary bone OS within the radiation field 19 years after the initial MM diagnosis [10]. There are no other cases in the literature of concurrent MM and primary bone OS.

In this case, the preliminary workup revealed both radiographic skeletal lesions and abnormal lab results that were consistent with the initial diagnosis of MM. Further radiographic investigations revealed additional lesions, originally found in the right hip, and later found in the lungs. Initially, the lesions in the right hip were attributed to the history of MM and the mass in the right pelvis was thought to be related to heterotopic bone from a previous right hip trauma and prosthesis. However, the correct diagnosis of OS was not given to the right pelvic mass until the metastatic nodules in the lung were definitively identified as histologically consistent with OS.

In this case, lung biopsy findings were necessary to confirm the diagnosis of metastatic high-grade osteogenic sarcoma, and the mass in the right hip with the sclerotic “sunburst” radiographic pattern was given a presumptive diagnosis of primary OS. In MM, sclerotic “sunburst” lesions, as opposed to the typical lytic “punched-out” lesions, have been reported, though rarely [4]. This suggests that further study and investigation may be warranted in MM patients presenting with sclerotic bone lesions. Additionally, further research is needed to determine the true prevalence of concurrent presentation of MM and primary bone OS.

## Conclusions

Given the prevalence of MM and OS in geriatric patients, it is important to consider a concurrent diagnosis of these two entities when confronted with a solitary densely sclerotic bone lesion in the midst of diffusely mottled lytic bone, typically seen in MM.

## References

[1] Kazandjian D (2016). Multiple myeloma epidemiology and survival: a unique malignancy. Semin Oncol.

[2] Edwards CM, Zhuang J, Mundy GR (2008). The pathogenesis of the bone disease of multiple myeloma. Bone.

[3] Kyle RA, Gertz MA, Witzig TE (2003). Review of 1027 patients with newly diagnosed multiple myeloma. Mayo Clin Proc.

[4] Ghosh S, Wadhwa P, Kumar A (2011). Abnormal radiologic features in a multiple myeloma patient: a case report and radiological review of myelomas. Dentomaxillofac Radiol.

[5] Mirabello L, Troisi RJ, Savage SA (2009). Osteosarcoma incidence and survival rates from 1973 to 2004: data from the Surveillance, Epidemiology, and End Results Program. Cancer.

[6] Angulo P, Kaushik G, Subramaniam D (2017). Natural compounds targeting major cell signaling pathways: a novel paradigm for osteosarcoma therapy. J Hematol Oncol.

[7] Bielack SS, Hecker-Nolting S, Blattmann C (2016). Advances in the management of osteosarcoma. F1000Research.

[8] Klein MJ, Siegal GP (2006). Osteosarcoma: anatomic and histologic variants. Am J Clin Pathol.

[9] Akiba T, Ujiie H, Takasaki N (1994). Endobronchial metastasis from a primary uterine osteosarcoma in a patient with multiple myeloma: report of a case. Surg Today.

[10] Vukmirović S, Jovanović V, Atanacković M (1994). Postradiation osteosarcoma in a patient with multiple myeloma. Srp Arh Celok Lek.

